# Host resources and parasite traits interact to determine the optimal combination of host parasite‐mitigation strategies

**DOI:** 10.1002/ece3.11310

**Published:** 2024-06-19

**Authors:** Andrew D. Dean, Dylan Z. Childs, Yolanda Corripio‐Miyar, Mike Evans, Adam Hayward, Fiona Kenyon, Luke McNally, Tom N. McNeilly, Robin J. Pakeman, Amy R. Sweeny, Daniel H. Nussey, Amy B. Pedersen, Andy Fenton

**Affiliations:** ^1^ Institute of Infection, Veterinary and Ecological Sciences University of Liverpool Liverpool UK; ^2^ School of Biosciences The University of Sheffield Sheffield UK; ^3^ Department for Disease Control Moredun Research Institute Penicuik UK; ^4^ The University of Edinburgh Royal (Dick) School of Veterinary Studies Roslin UK; ^5^ Institute of Ecology and Evolution, School of Biological Sciences University of Edinburgh Edinburgh UK; ^6^ The James Hutton Institute Aberdeen UK

**Keywords:** helminth, host condition, host nutrition, host resources, immune response, parasite life history, resistance, tolerance

## Abstract

Organisms have evolved diverse strategies to manage parasite infections. Broadly, hosts may avoid infection by altering behaviour, resist infection by targeting parasites or tolerate infection by repairing associated damage. The effectiveness of a strategy depends on interactions between, for example, resource availability, parasite traits (virulence, life‐history) and the host itself (nutritional status, immunopathology). To understand how these factors shape host parasite‐mitigation strategies, we developed a mathematical model of within‐host, parasite‐immune dynamics in the context of helminth infections. The model incorporated host nutrition and resource allocation to different mechanisms of immune response: larval parasite prevention; adult parasite clearance; damage repair (tolerance). We also considered a non‐immune strategy: avoidance via anorexia, reducing intake of infective stages. Resources not allocated to immune processes promoted host condition, whereas harm due to parasites and immunopathology diminished it. Maximising condition (a proxy for fitness), we determined optimal host investment for each parasite‐mitigation strategy, singly and combined, across different environmental resource levels and parasite trait values. Which strategy was optimal varied with scenario. Tolerance generally performed well, especially with high resources. Success of the different resistance strategies (larval prevention or adult clearance) tracked relative virulence of larval and adult parasites: slowly maturing, highly damaging larvae favoured prevention; rapidly maturing, less harmful larvae favoured clearance. Anorexia was viable only in the short term, due to reduced host nutrition. Combined strategies always outperformed any lone strategy: these were dominated by tolerance, with some investment in resistance.

Choice of parasite mitigation strategy has profound consequences for hosts, impacting their condition, survival and reproductive success. We show that the efficacy of different strategies is highly dependent on timescale, parasite traits and resource availability. Models that integrate such factors can inform the collection and interpretation of empirical data, to understand how those drivers interact to shape host immune responses in natural systems.

## Graphical abstract

We present a mathematical model of within‐host, immune‐parasite dynamics. We investigate how host nutrition, parasite life‐history and immune strategies interact to determine host outcomes. We find that hosts do best when prioritising tolerance of infection by repairing damage, combined with a low level of parasite resistance.

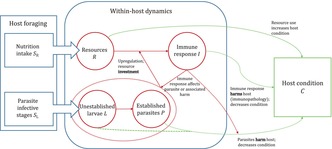

## 1 INTRODUCTION

Parasitic helminths (worms) are ubiquitous, have negative health and economic consequences for humans and domestic animals and negatively impact the health and population dynamics of wild animals (Bethony et al., 2006; Grenfell & Dobson, 1995; Hudson et al., 1998; Pedersen & Greives, 2008). Hosts have evolved diverse strategies to maintain fitness in the face of infection, the efficacy of which depends on numerous factors (Sears et al., 2011), including parasite identity (Budischak et al., 2018) and host nutritional status (Clough et al., 2016). The complexity arising from the interaction of these various factors can cloud understanding of why hosts adopt the strategies they do to combat parasitic infections, or the consequences of those strategies for the host and their parasites.

Parasitic helminths can exert costs on the host in a variety of ways. Many helminths infect via free‐living larval stages from the environment, which often enter the host through oral ingestion or skin penetration (Bethony et al., 2006). This infection process can cause significant damage to host tissue as larvae migrate through the host seeking their optimal location, often the gastrointestinal (GI) tract, where they moult into adult worms (Balic et al., 2000; Bethony et al., 2006). For example, larvae of several species of nematode subcutaneously infect mice and migrate via the airways to the small intestine, causing haemorrhage and inflammation in the lungs (Chen et al., 2012; Enobe et al., 2006). Established adult parasites then feed on host tissue such as blood or the gut lining, thus diminishing host condition, the severity of which would tend to increase with the burden of infection (Balic et al., 2000; Bethony et al., 2006; Coop & Holmes, 1996; Holmes, 1987). For example, higher parasite faecal egg counts (generally assumed to correlate with parasite burden) have been shown to correlate with body mass loss in Soay sheep (Hayward et al., 2014) and wild horses (Debeffe et al., 2016), with high parasite burdens being implicated in mortality in sheep (Gulland, 1992), whereas anthelmintic treatment has been shown to increase body condition, growth rate and survival in white‐footed mice (Vandegrift et al., 2008).

The deleterious effects of both invading parasitic larvae and established adult worms provide evolutionary pressure for the host species to develop strategies to combat them (Best et al., 2008; Lochmiller & Deerenberg, 2000; Read et al., 2008; Sorci, 2013). These strategies fall into three broad categories: infection avoidance, parasite resistance or disease tolerance. Infection avoidance is any pre‐emptive strategy involving a host changing its behaviour in order to minimise contact with parasite infective stages. One well‐documented strategy in the context of GI parasites is anorexia, hypothesised to reduce ingestion of parasite infective stages by reducing foraging or selectively grazing to avoid faeces; in the case of directly transmitted parasites, hosts may avoid contact with infected individuals (Adelman & Hawley, 2017; Ayres & Schneider, 2009; Ezenwa et al., 2022; Hite et al., 2020; Kyriazakis et al., 1998; Rao et al., 2017). Parasite resistance involves the host's immune system directly targeting its parasites, either larval or adult stages, to reduce infection via parasite killing and/or expulsion (Balic et al., 2000; Balic et al., 2002; Grencis, 2015; McRae et al., 2015; Reynolds et al., 2012). Lastly, disease tolerance does not involve the host targeting parasites; rather, the host mitigates and repairs damage caused by infection, without directly affecting the parasite itself (Kutzer & Armitage, 2016; Medzhitov et al., 2012; Råberg et al., 2007; Råberg et al., 2009; Read et al., 2008; Sorci, 2013). Understanding the contexts that affect the relative success of these different strategies, and the consequences to the host, remain major conceptual and logistical challenges, yet are fundamental to understanding how hosts maintain health and fitness in the face of helminth infection and to the development of effective treatments for humans and livestock.

It is well known that mounting an effective immune response to clear parasites, whether through killing or expulsion, is energetically costly (Lochmiller & Deerenberg, 2000; Sykes & Coop, 2001), and often comes with associated immunopathological damage (e.g., due to inflammation) (Graham et al., 2005; Sears et al., 2011). In helminth infections, resistance mechanisms generally target larvae as they migrate through host tissue, thus preventing tissue damage and parasite establishment (Balic et al., 2002; Esser‐von Bieren et al., 2013; Meeusen & Balic, 2000; Obata‐Ninomiya et al., 2013), while established adult infections are often tolerated, for example via repairing the associated damage to the GI tract (King & Li, 2018; Motran et al., 2018; Yap & Gause, 2018). It is generally assumed that the main benefit of a tolerance strategy is the absence of immunopathology, as the immune response needed to clear a large, multicellular adult helminth would likely cause severe immunopathology; hence, we would expect strong evolutionary pressure for a less harmful tolerance response (Allen & Wynn, 2011; Díaz & Allen, 2007; Sears et al., 2011). A tolerance response, though, also favours the parasite, as infection burden is not directly affected, allowing for chronic infections with greater opportunity for reproduction. This may lead to selection for parasite traits which promote tolerance, such as reduced (adult) virulence (King & Li, 2018; Motran et al., 2018; Sears et al., 2011; Yap & Gause, 2018). However, tolerance mechanisms are not without cost, as they can require a significant energetic input (Ayres & Schneider, 2012). They also carry a population‐level cost, in that higher parasite burdens presumably result in higher production of infective stages, thus increasing parasite transmission potential across the wider host population (Adelman & Hawley, 2017; Henschen & Adelman, 2019).

Understanding and predicting the consequences for the host of adopting different parasite mitigation strategies involves an assessment of the potentially complex interplay between parasite‐induced damage, immune‐induced damage and the energetic costs of mounting the response (Sykes & Coop, 2001). Fundamental to this is the role that host nutrition plays in mediating the balance between the costs and benefits of mounting any given control response. A substantial body of work has investigated the role of nutrition and diet in mounting an effective immune defence (Becker et al., 2018; Coop & Holmes, 1996; Cressler et al., 2014; Pedersen & Greives, 2008; Sykes & Coop, 2001), and, more recently in tolerating infection (Budischak & Cressler, 2018). In general, better resourced hosts can more readily withstand infection and/or mount an effective resistance response (Koski & Scott, 2001; Sykes & Coop, 2001). For example, dietary‐supplemented wood mice were better able to resist infection by the helminth *Heligmosomoides polygyrus* and maintained better body condition (Sweeny et al., 2021), whereas protein‐deficient laboratory mice had decreased intestinal barrier function (an indicator of tolerance) (Clough et al., 2016). In recent years, an increasing number of studies have begun to focus more specifically on the effect of diet on resistance versus tolerance (Budischak & Cressler, 2018; Kutzer & Armitage, 2016). When tree frogs on different diets were exposed to skin‐penetrating gut nematodes, resource‐poor hosts were successfully penetrated by a greater number of parasites, produced higher levels of antibodies and lost weight; parasites had a higher establishment rate in the guts of well‐fed hosts, but those hosts were able to maintain body mass in the face of infection (Knutie et al., 2017). A similar experiment involving *Drosophila melanogaster* exposed to the bacterium *Providencia rettgeri* found that a high‐sugar diet improved resistance and fecundity and reduced mortality compared to a low‐sugar one. However, the relationship between bacterial load and host fecundity was the same on both diets, that is, tolerance as measured by host mortality decreased on the low‐sugar diet, but not tolerance as measured by host fecundity (Howick & Lazzaro, 2014).

While previous work has assessed the relative benefits of different parasite mitigation strategies (e.g., resistance vs. tolerance), it remains an open question how host resource levels influence the health consequences of the host in adopting different strategies and how this is affected by different environmental conditions and parasite life‐history scenarios. Here, we investigate these questions by developing a mathematical model of within‐host interactions between a macroparasite (helminth) infection and alternative immune‐ and non‐immune‐mediated parasite mitigation strategies (e.g. avoidance, resistance, tolerance), while explicitly accounting for host resource acquisition and utilisation, and the balance of harm caused by the parasites and any immunopathology. Using this model, we evaluate how within‐host interactions between resource levels, host response and parasite traits combine to determine host condition, thereby influencing a host's optimal parasite mitigation strategy over both the short and long term.

## 2 METHODS

### 2.1 Model structure

We developed a general model of within‐host parasite‐resource‐immune interactions, building on previous work on microparasite infections (Budischak & Cressler, 2018; Cressler et al., 2014), to consider an individual host infected by a macroparasite (helminth), which infects via free‐living environmental stages. Although inspired by GI helminths in herbivore hosts, usually infecting via ingestion, the only species‐specific trait incorporated in the model is that the parasite undergoes a maturation phase after infection but does not replicate within the host. We modelled these within‐host dynamics via the coupled differential equations:

(2.1) $$\frac{dR}{dt}=\frac{S_{R}}{1+k_{A}\left(L+P\right)}-\mathrm{rR}-\frac{{\mathrm{cf}}_{I}R}{1+{\mathrm{vf}}_{I}R},$$

(2.2) $$\frac{dI}{dt}=\frac{f_{I}R}{1+{\mathrm{vf}}_{I}R}-\mathrm{lI},$$

(2.3) $$\frac{dL}{dt}=\frac{S_{L}}{1+k_{A}\left(L+P\right)}-\left(g+d_{L}+k_{L}I\right)L,$$

(2.4) $$\frac{dP}{dt}=\mathrm{gL}-\left(d_{P}+k_{P}I\right)P,$$

(2.5) $$\frac{dC}{dt}=\left[\frac{\mathrm{arR}}{1+\mathrm{bC}}-w-\frac{h_{L}L+h_{P}P}{1+k_{T}I}-h_{I}I\right]\Theta \left(C\right).$$

**FIGURE 1:**
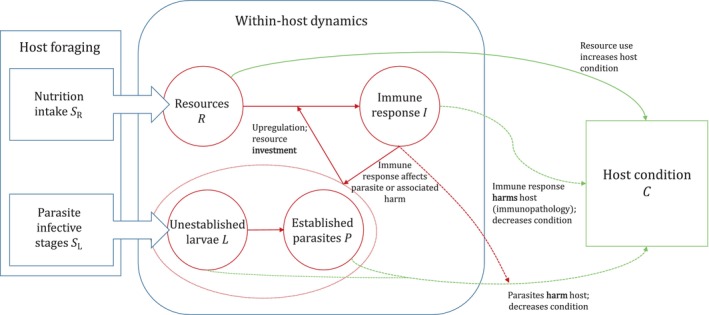
Schematic representation of the model represented by Equations ((2.1), (2.2), (2.3), (2.4), (2.5)). Host foraging results in a constant intake of resources $S_{R}$ and larval parasite infective stages $S_{L}$. Parasite larvae L mature into established adults P and interact with the immune response I, causing upregulation of a parasite‐specific response. Resources are used to produce the immune response and maintain host condition C. Host condition deteriorates due to parasite damage and immunopathological harm. Immune responses can affect either parasite larvae or adults (resistance) or repair parasite‐induced damage (tolerance). The (non‐immune‐mediated) avoidance strategy is modelled as anorexia, where an individual reduces time spend foraging, and hence reduces both the intake of nutrients and parasite infective stages.

$R\left(t\right)$ represents the within‐host resource pool (i.e. resources available to the host) at time t. $I\left(t\right)$ is the magnitude of the immune response that is upregulated in response to the presence of the parasite. $L\left(t\right)$ and $P\left(t\right)$ are the larval and adult parasite burdens, and $C\left(t\right)$ is a measure of host condition. A schematic diagram of the model system is presented in Figure 1. Variables and parameters are defined in Table 1, along with baseline parameter values used in our analyses.

**TABLE 1:** (a) System variables and their units. (b) System parameters, their units and default values used in simulations.

a. Variable	Symbol	Units
Within‐host resource availability	R	Mass vol.^−1^
Immune response density	I	Mass vol.^−1^
Within‐host larval parasite density	L	Num. vol.^−1^
Within‐host adult parasite density	P	Num. vol.^−1^
Host condition	C	*Dimensionless*
Time	t	Day

b. Parameter	Symbol	Units	Values
Resource availability	$S_{R}$	Mass vol.^−1^ day^−1^	0–5
Host resource consumption (non‐immune processes)	r	Day^−1^	1
Investment in immune response	c	*Dimensionless*	*Optimised*
Initial proportion of resources allocated to constitutive immune response $I_{0}$	p	*Dimensionless*	0.01
Immune system upregulation resource consumption factor	q	Vol.^2^ num.^−1^ mass^−1^ day^−1^	0.1
Coefficient of saturation in immune response production	v	Vol. day mass^−1^	0.5
Immune particle degradation	l	Day^−1^	0.1
Infection pressure (parasite infective stage ingestion)	$S_{L}$	Num. vol.^−1^ day^−1^	0.5, 2
Larval maturation into adults	g	Day^−1^	0.1,0.5
Larval mortality	$d_{L}$	Day^−1^	0.1
Adult parasite mortality	$d_{P}$	Day^−1^	0.02
Conversion rate of resource consumption into host condition	a	Vol. mass^−1^	2
Coefficient of diminishing returns of conversion of resources into host condition	b	*Dimensionless*	1
Loss of host condition	w	Day^−1^	1
Per capita larval harm	$h_{L}$	Vol. num.^−1^ day^−1^	0.2, 0.4, 0.6
Per capita adult parasite harm	$h_{P}$	Vol. num.^−1^ day^−1^	$0.8-h_{L}$
Immunopathological harm	$h_{I}$	Vol. mass^−1^ day^−1^	$0.25k_{\text{strategy}}+0.25k_{\text{strategy}}^{2}$, $\text{strategy}\in \left\{L,P\right\}$
Strength of anorexia strategy	$k_{A}$	Vol. num.^−1^	*Optimised*
Strength of prevention strategy	$k_{L}$	Vol. mass^−1^ day^−1^	$\frac{c}{1+0.5c}$
Strength of clearance strategy	$k_{P}$	Vol. mass^−1^ day^−1^	$\frac{c}{1+0.5c}$
Strength of tolerance strategy	$k_{T}$	Vol. mass^−1^	$\frac{c}{1+0.5c}$

The host is assumed to have a constant (if not avoiding parasites via anorexia) supply of resources $S_{R}$, obtained through foraging. Resources for non‐immune processes (growth, metabolism etc.) are allocated at rate r. The third term on the right‐hand side of (2.1) represents the diversion of resources to the host immune response; the first term on the right‐hand side of (2.2) thus represents the consequent production of that immune response, where c is the unit resource investment required. We assumed that there are two processes that combine to determine the magnitude of the immune response. Firstly, standing constitutive immunity, which we represent by setting the initial value of the immune response to be non‐zero (cf. Appendix A). Secondly, an inducible, parasite‐specific response that is upregulated through contact with the infection; we represent this contact, or ‘immune stimulation’, as

(2.6) $$f_{I}\left(L,P,I\right)≔q\left(L+P\right)I.$$

Stimulation of the parasite‐specific, inducible response therefore occurs proportionally to the contacts between the current immune response I and the total parasite burden L+P, with rate q. Although immune stimulation is unbounded, we assumed an upper limit to the actual production of the immune response and therefore set immune production to be a saturating function of immune stimulation $f_{I}$, as seen in (2.1–2.2). Here, the constant v determines how quickly immune production saturates with respect to stimulation; if v=0, immune production is equal to immune stimulation. The immune response decays at a constant rate l.

We assumed that the host was constantly exposed to parasite infective stages and hence the larval parasite load had constant input $S_{L}$. If infection is via ingestion, for example by grazing on contaminated pasture, both $S_{R}$ and $S_{L}$ are proportional to the foraging effort. Note that other infection mechanisms, such as skin penetration, would decouple the two rates; in such an instance, anorexia would not reduce exposure and so cannot function as a parasite avoidance strategy. Upon infection, parasite infective larval stages mature into adults at rate g. Larval and adult parasites have natural clearance rates $d_{L}$ and $d_{P}$.

Equation (2.5) determines host condition $C\left(t\right)$, which acts as a metric of host fitness (e.g. higher condition increases survival, offspring health, mating opportunities), combining the effects of the host's nutritional state and parasite burden. This variable may be physically represented by a metric of invested physical resources such as scaled body mass, coat health and fat reserves, or some combination of these. However, such measures may not always be as responsive to parasite damage as implied by Equation (2.5). This issue may be overcome by also incorporating specific markers of harm (tissue damage). For an intestinal parasite, an appropriate metric may be one measuring gastrointestinal condition, such as faecal calprotectin as a marker of gut inflammation (implicated in sheep (Váradyová et al., 2017); validated in humans (Jukic et al., 2021)), blood in faeces as a marker of gut damage (Jiminez et al., 2015), or the presence of microbial products and associated antibodies in the blood as a marker of gut permeability (González‐González et al., 2019). We assumed that processed resources (cf. the second term on the right‐hand side of (2.1)) are converted into host condition with diminishing returns, that is, the same increase in condition requires more resources for a well‐conditioned host than a poorly conditioned one. This is represented by the first term on the right‐hand side of (2.5); a represents the baseline conversion of processed resources into condition, while b determines how rapidly the resource requirement increases with condition. Hence, a non‐zero value of b ensures that condition cannot increase indefinitely. We imposed a constant loss of condition w, representing energetic requirements such as metabolism, movement and maintenance of body temperature. Damage due to infection was assumed to arise through the combined effect of harm caused by the parasite (with per capita damage coefficients $h_{L}$ and $h_{P}$ for larval and adult parasites respectively, thus incorporating damage done during the larval tissue migration phase and by adults feeding on host tissue) and harm caused by the immune response (immunopathology such as inflammation, with coefficient $h_{I}$). If at any point C=0, the host dies, as determined by the Heaviside step function $\Theta \left(x\right)$ with $\Theta \left(0\right)=0$,

(2.7) $$\Theta \left(x\right)=\left\{\begin{array}{ccc} 1, & \; & x>0,\\ 0 & \; & x\leq 0. \end{array}\right.$$

In formulating the model (2.1)–(2.5), we assumed that parasite numbers remained sufficiently low that neither larval nor adult parasites are subject to density‐dependent limitation. Such effects are likely unimportant to an individual host under low to moderate infection pressure but would need to be included if scaling our model to the population level, in order to fully capture nonlinear feedback in transmission between individuals. We also assumed that the state of both the environment and the host was constant over the timescale of the simulations. Over the longer term, such variation is likely to be important; for example, the transition from summer to winter reduces resource availability and increases host energetic demands due to less clement weather, and pregnant or suckling females must expend significant resources on their young. The effects of such variation can be inferred by considering the different parameter values used in our simulations.

### 2.2 Alternative parasite‐mitigation strategies

We assumed the host can combat infection through one of four strategies, where the parameters $k_{A},k_{L},k_{P},k_{T}$ determine the strength of each strategy (i.e., a higher value of k yields an increased effect):

- *Avoidance: Parasite‐related anorexia* The host reduces its resource intake (the first term in (2.1); strength $k_{A}$) to reduce exposure to new parasite infective stages (the first term in (2.3)). Note that this is a pre‐infection strategy and does not utilise an immune response; hence, anorexia comes with no associated immunopathology, although the host's ability to maintain condition is hampered by the decrease in resource availability. As this strategy is not immune mediated, we set q=0 and fix I=0, so there is no explicit immune response. The extreme version of this strategy is starvation ($k_{A}=\infty$), in which the host has zero intake of both resources and infective stages (if infection is via ingestion). If infection is not via ingestion, this strategy has no benefit.
- *Resistance response 1: Prevention of larval parasite establishment* The host mounts a resistance response whereby the immune system targets larvae before establishment, increasing their mortality (the final term in (2.3); strength $k_{L}$). We assumed that such an immune response induces a certain level of immunopathological harm to the host ($h_{I}>$ 0).
- *Resistance response 2: Clearance of adult parasites* An alternative resistance response involves the immune system targeting established, adult parasites, increasing their mortality or expulsion rate (the final term in (2.4); strength $k_{P}$). Again, we assumed such a response induces immunopathological harm ($h_{I}>$ 0).
- *Tolerance response: Immune‐mediated damage mitigation* Here, the immune system makes no attempt to reduce the parasite burden. Instead, the host mitigates the harm caused by the parasites (the third term in (2.5); strength $k_{T}$). We assumed such a response to have no associated immunopathology ($h_{I}$ = 0). Note that this version of tolerance is immune‐mediated, that is, upregulated in response to infection. The term tolerance may also be used to describe damage repair without such upregulation, that is, as a response to the damage itself rather than an explicit response to the parasite. Such damage repair occurs through the direct conversion of resources into condition; in our model, this is implicitly included in the first term on the right‐hand side of (2.5).

**FIGURE 2:**
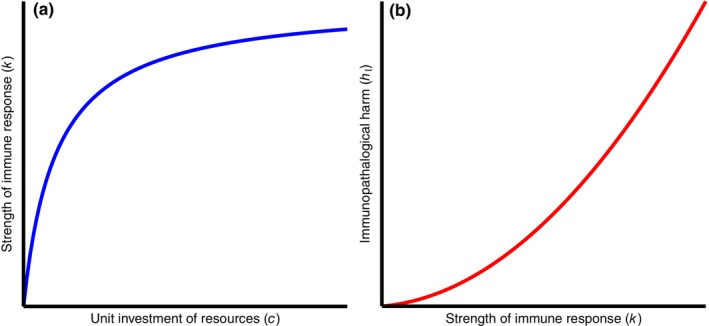
Sketch of immune parameter relationships. (a) Strength of immune response k is a saturating function of the resource investment c. (b) Immunopathological harm $h_{I}$ is a quadratically increasing function of the strength of the immune response k.

We assumed throughout that a more energetically expensive response has a stronger effect on the parasite, and so set the strength of each strategy (the k parameters in (2.1)–(2.5)) to increase with the unit investment c of the immune response. As an infinitely strong response is biologically unfeasible, we also imposed an upper limit to the achievable strength of each *immune‐mediated* strategy (i.e. prevention of larval establishment $k_{L}$, clearance of adult parasites $k_{P}$ and tolerance $k_{T}$, but not anorexia) via the following saturating relationship between the strength of the immune response and its unit investment,

(2.8) $$k_{\text{strategy}}=\frac{k_{0}c}{1+k_{1}c},\;\text{strategy}\in \left\{L,P,T\right\},$$

as illustrated in Figure 2a. This relationship (2.8) also ensures that if the unit investment c is zero then the immune response has zero strength and has no effect on the parasite. Note that (2.8) does not apply to the anorexia strategy, as it is not immune‐mediated. Rather, $k_{A}$ is unbounded, and represents both the strength of the strategy and its indirect resource cost due to the reduction in ingestion rate. When $k_{A}$ is sufficiently large, the host is effectively starving.

We also assumed that a stronger immune response causes increasingly severe immunopathology. This provided another check against a host simply investing heavily in clearance, a situation we deemed biologically unfeasible due to the large and complex nature of a helminth. Thus, we set

(2.9) $$h_{I}=h_{0}k_{\text{strategy}}+h_{1}k_{\text{strategy}}^{2},\;\text{strategy}\in \left\{L,P\right\},$$

as shown in Figure 2b. Hence, the immunopathological harm increases quadratically with the strength of the immune response for the two resistance strategies (prevention and clearance), the principal advantage of the avoidance and tolerance strategies being the absence of immunopathology.

### 2.3 Model analyses

We used this model to explore the outcomes of each of the four strategies described above over a range of environmental conditions and parasite traits. For simplicity, we initially assume that the host adopts only a single strategy at a time, and so we investigated the effect of each strategy in isolation by setting the strengths of the other three strategies to zero.

We first evaluated predicted dynamics by integrating the system (2.1)–(2.5), starting from a parasite‐free state (cf. Appendix A for details) over one ‘season’ lasting 90 days ($t\in \left[0,90\right]$; see Appendix B for a discussion of parameter selection). This time period was chosen to explore relatively long‐term dynamics while assuming that environmental and demographic factors remain relatively constant. We evaluated the consequences of a range of levels of investment c in each parasite‐mitigation strategy in turn, under contrasting levels of high or low resource availability $S_{R}$. In these simulations, the level of immune investment remained constant throughout the duration.

We then used the R function optim to calculate the optimal investment value for each strategy in turn, determined by maximising mean host condition (as a measure of fitness) over a specified time period. We did so first in the short term (1 week; $t\in \left[0,7\right]$) and then in the long term (one season (90 days); $t\in \left[0,90\right]$). This was repeated for increasing levels of resource availability $S_{R}$, different adult‐to‐larval per capita ratios of harm (with $h_{L}+h_{P}$ fixed at 0.8 to facilitate comparisons) and different parasite maturation rates g, in order to compare host fitness consequences for various environmental conditions and parasite traits. We also carried out a sensitivity analysis by exploring the effects of different values of several key parameters; the results of this are presented in Figures S8–S10 and show that outcomes remain qualitatively very similar.

### 2.4 Combined strategies

In reality, organisms are not limited to a single strategy, but utilise a combination of strategies against their parasites (Budischak et al., 2018; DeSimone et al., 2018; Read et al., 2008). We therefore expanded the previous analyses to investigate the effects on host condition of combining all three immune‐mediated strategies, in differing proportions (for simplicity, we omitted anorexia from this analysis, as it was a non‐viable long‐term strategy; see below). We assumed that each strategy had the same strength k, but the overall immune response was divided between larval prevention, adult clearance and tolerance via the proportions $\nu_{L},\nu_{P},\nu_{T}$, respectively, which were constrained so that

(2.10) $$\nu_{L}+\nu_{P}+\nu_{T}=1,$$

and

(2.11) $$0\leq \nu_{\text{strategy}}\leq 1,\;\text{strategy}\in \left\{L,P,T\right\}.$$

Thus, a lone strategy could be represented by setting one ν parameter to unity, forcing the other two to be zero. Given these assumptions, we rewrote (2.1)–(2.5) as

(2.12) $$\frac{dR}{dt}=S_{R}-\mathrm{rR}-\frac{{\mathrm{cf}}_{I}R}{1+{\mathrm{vf}}_{I}R},$$

(2.13) $$\frac{dI}{dt}=\frac{f_{I}R}{1+{\mathrm{vf}}_{I}R}-\mathrm{lI},$$

(2.14) $$\frac{dL}{dt}=S_{L}-\left(g+d_{L}+\nu_{L}\mathrm{kI}\right)L,$$

(2.15) $$\frac{dP}{dt}=\mathrm{gL}-\left(d_{P}+\nu_{P}\mathrm{kI}\right)P,$$

(2.16) $$\frac{dC}{dt}=\left[\frac{\mathrm{arR}}{1+\mathrm{bC}}-w-\frac{h_{L}L+h_{P}P}{1+\nu_{T}\mathrm{kI}}-h_{I}I\right]\Theta \left(C\right).$$

We also ensured that only resistance strategies contributed to immunopathology by rewriting (2.9) as

(2.17) $$h_{I}=h_{0}\left(\nu_{L}+\nu_{P}\right)k+h_{1}\left(\nu_{L}^{2}+\nu_{P}^{2}\right)k^{2}.$$

Note that, for simplicity, we have assumed that both resistance strategies contribute equally to immunopathology.

By concurrently optimising mean host condition over the investment c and two of the three ν parameters (with the third then determined by the constraints (2.10)–(2.11)), we were able to compare strategies in combination against those in isolation and investigate how the optimal proportion of immune response allocated to each of the three strategies varied with environment and parasite traits.

## 3 RESULTS

**FIGURE 3:**
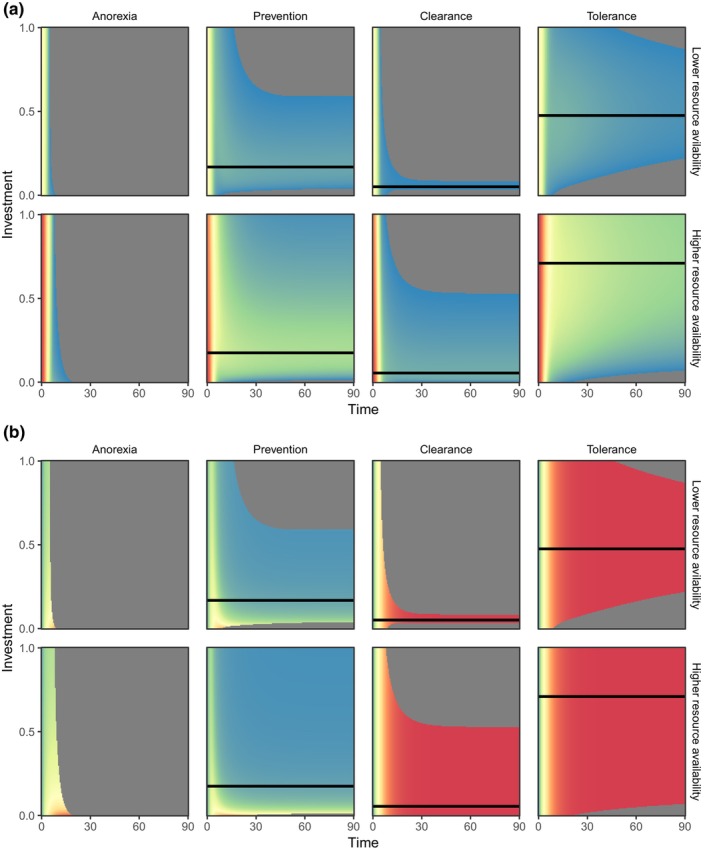
Comparative dynamics (time on x‐axis) of varying levels of investment (c, y‐axis) in each of the four parasite‐mitigation strategies (anorexia, larval parasite prevention, adult parasite clearance, tolerance) on (a) host body condition and (b) adult parasite burden, under two different values of resource availability; low ($S_{R}=3$; top rows) and high ($S_{R}=5$; bottom rows). Parasite larvae and adults are assumed to be equally harmful ($h_{L}=h_{P}=0.4$); parasite maturation rate is set at g=0.1; infection pressure is $S_{L}=2$. The black lines indicate the value of c that maximises mean host condition. The heat maps are scaled so values increase from blue to red; colours are normalised independently over each variable so that the scale is different for host condition than for mature parasite load. Grey represents a dead host ($C\left(t\right)=0$).

Both host condition and parasite burdens were predicted to be differentially impacted by the choice of host parasite mitigation strategy. Figure 3 shows model trajectories over time (x‐axis) for different levels of investment (c; y‐axis) in the four parasite‐mitigation scenarios considered (columns), under conditions of low resource availability (top rows) and high resource availability (bottom rows). The system variables presented are host condition ($C\left(t\right)$, Figure 3a) and adult parasite burden ($P\left(t\right)$, Figure 3b); the corresponding figures for host resource levels, immune response and larval parasite burden can be found in Figure S1. In all cases, initially parasite‐free hosts are exposed to infection, leading to a loss of condition as their parasite burden increases with time. Unsurprisingly, better‐resourced hosts (bottom rows for each variable) have higher condition and can survive for a broader range of immune investment than poorly resourced hosts. However, anorexia (first column) is not a viable long‐term strategy, as the host inevitably dies within ~20 days, even under high resource availability. Generally, preventing larval establishment (second column) and disease tolerance (fourth column) lead to higher host condition than adult parasite clearance, although tolerance requires a greater unit investment in the immune response than prevention or clearance. Adult clearance, however, is the most effective strategy for reducing parasite loads, and prevention of larval establishment is much better than tolerance, as the latter strategy does not impact parasites at all.

**FIGURE 4:**
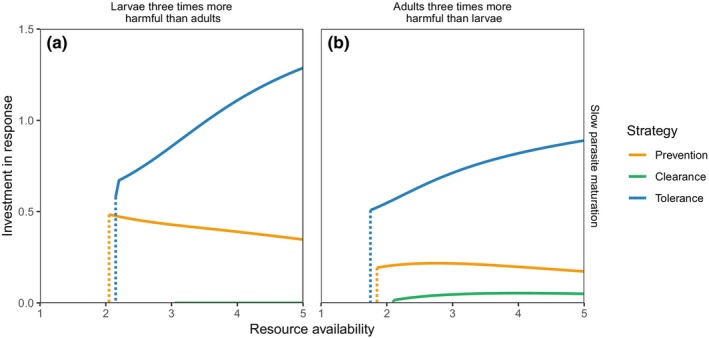
Short‐term maximum mean condition achievable over 1 week ($t\in \left[0,7\right]$), for each lone parasite‐mitigation strategy, over a range of resource availability levels, infection pressure $S_{L}=2$ and a relatively slow parasite maturation rate of g=0.1. (a) Larvae have higher per capita harm than adults ($h_{L}=0.6,h_{P}=0.2$). (b) Adults have higher per capita harm than larvae ($h_{L}=0.2,h_{P}=0.6$). Parasite maturation rate is set to be relatively slow at g=0.1. Data are plotted only for those parameter values for which the host survives; dashed vertical lines indicate the minimum value of $S_{R}$ for which the host survives (minimum‐resource survival threshold). The starvation strategy represents complete anorexia (zero resource intake, corresponding to $k_{A}=\infty$). In (a), any investment in the clearance strategy decreases host condition, i.e., the optimum investment is zero, making this equivalent to no strategy.

The host condition heat maps demonstrate there is generally an optimum investment value for each strategy that yields the highest mean condition over the simulation period (black lines in Figure 3). Figure 4 shows the mean condition achievable by these optimal investments in each strategy over the short term (1 week), for a range of resource availability values $S_{R}$, and for two pairs of values of larval and adult parasite harm (constrained so that $h_{L}+h_{P}=0.8$). The corresponding investment c is plotted in Figure S2, and the final parasite burdens in Figure S3. Each strategy has a value of $S_{R}$ below which the host dies, indicated by dashed lines in Figure 4; we refer to this value as the minimum‐resource survival threshold. When adult parasites cause more per capita harm than larvae, tolerance is the best strategy, with little difference between the others (Figure 4b). However, when larvae are more harmful, prevention has a lower minimum‐resource survival threshold than tolerance (Figure 4a). In addition, anorexia in the form of complete starvation ($k_{A}=\infty$) is the best strategy by a small margin if resources are sufficiently plentiful. Resource availability affects the starvation strategy because it determines the initial condition of the host (initial resources are $R\left(0\right)=S_{R}/r$; cf. Appendix A); more resources means the host is initially in better condition and can therefore survive starvation for longer. Interestingly, when larvae are more harmful than adults, any investment in adult clearance decreases the mean host condition, and thus the optimal investment for this strategy is zero, equivalent to no strategy (cf. Figure S2a).

**FIGURE 5:**
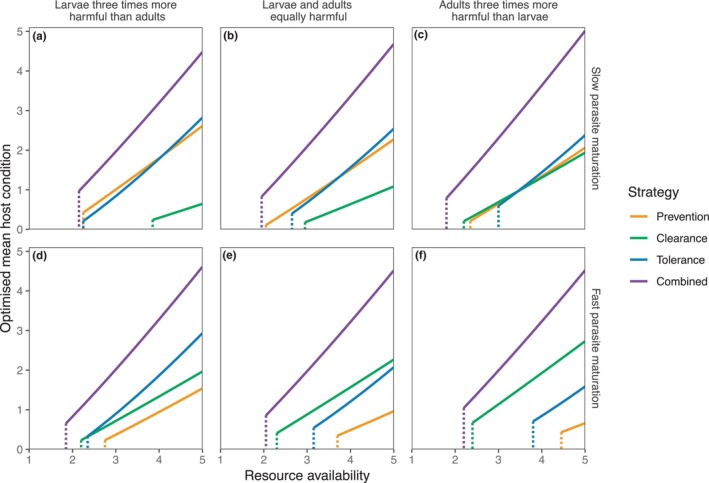
Long‐term maximum mean condition over one season ($t\in \left[0,90\right]$), for each parasite‐mitigation strategy, alone and combined, for a range of resource availability levels and infection pressure $S_{L}=2$. Left column: adults are less harmful than larvae ($h_{L}=0.6,h_{P}=0.2$). Centre column: adults and larvae are equally harmful ($h_{L}=h_{P}=0.4$). Right column: adults are more harmful than larvae ($h_{L}=0.2,h_{P}=0.6$). Top row: parasites mature relatively slowly (g=0.1). Bottom row: parasites mature relatively quickly (g=0.5). Data are plotted only for those parameter values for which the host survives; dashed vertical lines indicate the minimum value of $S_{R}$ at which the host survives (minimum‐resource survival threshold). The anorexia strategy or no strategy do not appear in any panel, as both choices lead to host death for these parameters over this time period.

We then explored the longer‐term results of maximising mean host condition over the course of one 90‐day season ($t\in \left[0,90\right]$) rather than 1 week. In this case, we considered three pairs of values of larval and adult parasite harm (again constrained so that $h_{L}+h_{P}=0.8$; Figure 5, columns) and two values of parasite maturation (g; Figure 5, rows). On this longer timescale, anorexia always led to host death, as hosts were not sufficiently well‐resourced to survive an entire season with a reduction in resource intake. Thus, anorexia is absent from Figure 5. Adopting no strategy at all was viable when infection pressure was low ($S_{L}=0.5$; Figure S6), but led to host death across all scenarios when infection pressure was high ($S_{L}=2$, consequently adopting no strategy is absent from Figure 5). For the remaining lone strategies, we saw a range of outcomes, dependent on the balance of resource levels, larval and adult harm and parasite maturation rate. When parasites matured slowly (Figure 5a–c), prevention and tolerance were similarly viable, although the minimum‐resource survival threshold for tolerance increased as adult parasites became relatively more harmful (Figure 5c). Conversely, clearance became less viable, both in terms of minimum‐resource survival threshold and host condition, as larvae increased in harm compared to adults (Figure 5c cf. Figure 5a). When parasites matured rapidly, prevention was always the least viable strategy (Figure 5d–f). Infection with more harmful adult parasites favoured a clearance strategy (Figure 5f), whereas tolerance became optimal when larvae were more harmful (Figure 5d). However, clearance had a lower minimum‐resource survival threshold than tolerance for all scenarios with rapidly maturing parasites and hence remained viable for lower resource levels in these cases.

**FIGURE 6:**
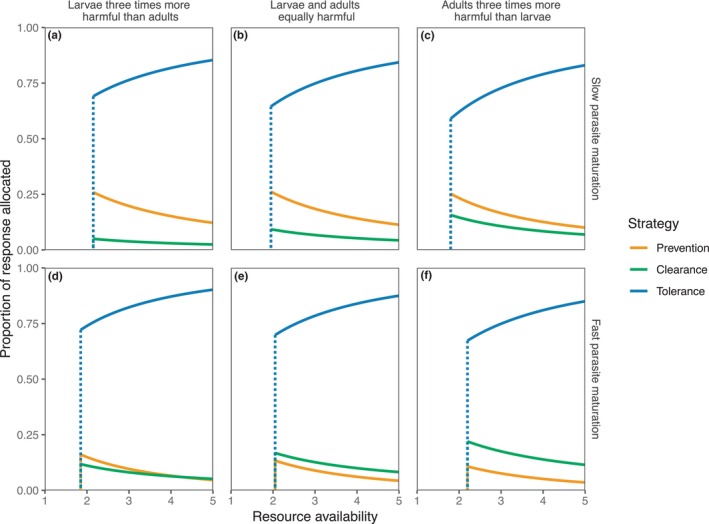
Proportion of the immune response allocated to each arm of the combined strategy depicted in Figure 5. Each line plots the value of the associated ν‐parameter that maximises mean host condition.

Allowing hosts to combine the three immune‐mediated strategies resulted in universally better outcomes for hosts than any strategy in isolation (Figure 5); mean host condition was approximately 50−400% greater for the combined strategy, and had lower minimum‐resource survival thresholds, than any lone strategy. In all scenarios explored, by far the greatest portion of the overall combined response was allocated to tolerance (Figure 6), and this proportion increased as resource availability increased. Notably, though, in no cases was a ‘pure’ tolerance response seen; the overall response always included some allocation to a resistance strategy. Slowly maturing parasites and those with more harmful larvae induced a greater allocation of immune responses to prevention, while more rapidly maturing parasites or those with more harmful adults induced greater clearance.

The parasite trait values considered here yield a two‐dimensional continuum of larval maturation time and adult‐to‐larval per capita ratio of harm. This can be summarised by considering overall more virulent adults (fast maturation, higher adult‐to‐larval ratio of harm) compared to overall more virulent larvae (slower maturation, lower adult‐to‐larval ratio of harm). In Figure 5, for example, panel (a) represents the overall most virulent larvae compared to adults (long‐lived larval stages, with high larval harm and low adult harm), with larval virulence decreasing, roughly speaking, as we progress through the panels to panel (f) representing the overall most virulent adults (short‐lived larval stages, with low larval harm and high adult harm). Correspondingly, prevention (targeting larvae) performs increasingly worse and clearance (targeting adults) performs increasingly better as we progress from panels (a) to (f). This same pattern can be seen in Figure 6, in which the amount of the immune response in a combined strategy that is devoted to prevention or clearance varies with the relative virulence of adults and larvae.

## 4 DISCUSSION

In recent years, tolerance has become widely accepted as a disease‐mitigation strategy in animals (Ayres & Schneider, 2012; Budischak & Cressler, 2018; Medzhitov et al., 2012; Read et al., 2008). Our model validates this shift in scientific understanding and shows that we would expect tolerance to often be preferred over resistance, although this is heavily dependent on the combination of parasite traits and resource availability (Figure 5). Moreover, a combined strategy, strongly weighted towards tolerance but also including some low‐level investment in resistance, universally outperformed all lone strategies. This is borne out in reality, where the type 2 immune response typically associated with helminth infections comprises both parasite killing and tissue repair (Allen & Sutherland, 2014; Coakley & Harris, 2020). The precise allocation of immune response between larval parasite prevention, adult parasite clearance and tolerance depended upon the scenario under consideration in a manner that corresponded to which of the lone strategies was more favourable. As such, we may expect to see significant variation in how hosts defend themselves against parasites, as well as variation in the consequences of adopting those different strategies, dependent upon environmental, parasite and individual host circumstances.

Resistance mechanisms were optimised by targeting the most virulent parasite life stage, with virulence comprising a combination of maturation rate (g) and direct harm ($h_{L}$ and $h_{P}$). Larval developmental time had a greater effect than harm in determining whether targeting adults or larvae is preferable; targeting rapidly maturing larvae is only weakly effective as they soon escape the immune response by transitioning to adults. This effect holds even though we assumed natural larval mortality to be considerably higher than that of adults throughout ($d_{L}=0.1$ compared to $d_{P}=0.02$). In ovine helminths, for example, maturation times range from 14–16 days (*Strongyloides papillosus*) to 8–12 weeks (*Fasciola* spp.) (European Medicines Agency, 2023); based on our findings, we may expect increased immune response to larvae at the higher end of this range.

In reality, larvae are often more virulent than adult parasites, as they migrate through host tissue in search of a suitable location to establish; this takes time and causes damage (Chen et al., 2012; Enobe et al., 2006). Furthermore, the immunopathology induced by attempting to clear adult parasites, given their generally large size, could be severely detrimental to the host (King & Li, 2018; Motran et al., 2018). Overall, we may expect to see resistance mechanisms preferentially targeting larvae, as the most harmful life‐cycle stage over adults, in line with theoretical predictions that hosts should resist more virulent parasites (Shudo & Iwasa, 2001). Indeed, immune responses can target larvae and adult parasites quite differently in sheep (Balic et al., 2000); for example, challenges with the abomasal (stomach) nematode *Haemonchus contortus* suggest that immune responses can be directed at either pre‐ or post‐establishment parasites (Balic et al., 2002). Furthermore, eosinophils are implicated in immune trapping or killing of helminth larvae infecting mice and sheep (reviewed in Meeusen & Balic, 2000); for example, it has been shown in mice that antibodies can trap migrating *Nippostrongylus brasiliensis* larvae in the skin, preventing maturation, but that the same immune response does not contribute to adult worm expulsion (Obata‐Ninomiya et al., 2013). From the host's point of view, focusing resistance mechanisms on larvae has the dual benefits of limiting the majority of parasite‐induced damage and reducing established infections, whereas targeting adults only does the latter. Similarly, targeting parasites will reduce transmission more than targeting adults, thus reducing the population‐level burden as well as that of the individual.

Although it may in general be optimal to target larvae, adult helminths vary in their pathogenicity, particularly as a result of their feeding strategies. For example, intestinal cestodes such as *Moniezia expansa* in sheep passively absorb nutrients through their tegument and are associated with little evidence for intestinal pathology or marginal or no impacts on host bodyweight (Elliott, 1986). In contrast, the large quantities of blood lost at the feeding site of the sanguivorous nematode *H. contortus* can lead to an often fatal anaemia in small ruminants (Besier et al., 2016). Our results suggest that tolerance may be a better strategy against infecting *M. expanza*, and resistance against *H. contortus*. The consequences of resisting a virulent adult helminth can be seen in a study on African buffalo (Budischak et al., 2018). When buffalo parasite burdens were tracked over time, those that gained the blood‐feeding helminth *Haemonchus* were found to have elevated immune defences but lost body condition. In contrast, those that gained the less pathogenic parasite *Cooperia* gained condition and had increased survival and fecundity, suggesting that a tolerance strategy had been employed against this parasite. It may be that the higher virulence of *Haemonchus* compared to *Cooperia* provoked a resistance immune response, but the hosts suffered from both increased parasite damage and immunopathology, hence the loss in condition.

Although host condition was predicted to increase with resource availability for all strategies, this was most marked for tolerance, which often exhibited the steepest gradient (the highest increase in host condition for a unit increase in resources) and achieved higher conditions than other lone strategies as resource availability increased. However, the minimum resource threshold below which the host dies was almost always higher for tolerance than for at least one of the resistance strategies, particularly for more virulent adult parasites (Figure 5e–f). We see in Figure S4 that the unit investment is much higher for the tolerance strategy, suggesting that energetic demands for tolerance are greater than other strategies. These findings complement empirical studies in various host organisms which have shown that tolerance requires adequate nutrition (Clough et al., 2016; Howick & Lazzaro, 2014; Knutie et al., 2017; Sweeny et al., 2021), that resource‐poor tree frogs had higher antibody levels (Knutie et al., 2017), and also suggests that tolerance is a poor strategy against highly virulent parasites (Sears et al., 2011; Shudo & Iwasa, 2001). Theory suggests that hosts with a slow pace of life should adopt a tolerance strategy, as such an organism should prioritise long‐term survival over short‐term reproduction (Sears et al., 2011). If tolerance has a high minimum‐resource survival threshold, as predicted here, then adopting such a strategy could make a host vulnerable to severe infection in times of reduced resource availability, as seen in the winter mortality of Soay sheep with high parasite burdens (Gulland, 1992).

Although we were primarily motivated by parasites that feed on host tissue (Balic et al., 2000; Bethony et al., 2006; Coop & Holmes, 1996; Holmes, 1987), another possibility is that a parasite instead steals resources directly. Mathematically, this would require a resource theft term in (2.1), which, in theory, the immune response could also ameliorate. Existing theory on microparasites (which replicate within the host) suggests that such a mechanism would diminish the resources available for an immune response (Cressler et al., 2014). However, the corresponding reduction in direct harm may make up for the reduction in condition due to reduced resources, making tolerance an even more attractive option. Conversely, parasites that replicate within the host and cause direct damage are likely to have increased virulence, suggesting that resistance should be preferred in such a case (Sears et al., 2011; Shudo & Iwasa, 2001).

In the present work, we have defined the tolerance response as damage repair (as opposed to behavioural tolerance (Adelman & Hawley, 2017)) and focused on immune‐mediated tolerance, i.e., damage repair that is upregulated by interactions between the immune system and the parasite. Non‐immune‐mediated tolerance is that which is a direct response to damage itself, irrespective of the parasites causing it; in our model, this aspect is implicitly incorporated into the first term in Equation (2.5), representing the host allocating its resources to increase its condition. This implicit tolerance contributes to the success of every strategy, and is part of the reason why greater resource availability increases host condition. We also note that we only explicitly considered tolerance to parasite‐inflicted damage; immune‐mediated tolerance mechanisms may equally well be applied to immunopathology. Indeed, the combination of tolerance for immunopathology and parasite resistance may be very effective. Similarly, the reliance of tolerance on resource availability suggests that behavioural feedback such as increasing resource intake (increased foraging) to promote tolerance, as seen in tree frogs (Knutie et al., 2017) and blue tits (Tripet & Richner, 1997), is a viable combination of strategies, although this could increase exposure to parasites that infect their hosts through ingestion. Furthermore, although we have not here found it to be viable as a lone strategy over long time periods, behavioural avoidance through anorexia can affect the efficacy of a tolerance or resistance strategy in *D. melanogaster* (Ayres & Schneider, 2009), perhaps by being immunostimulatory (Hite et al., 2020; Sykes & Coop, 2001), and so is worth investigating further as part of a mixed strategy.

As expected, adult parasite burdens were substantially higher when tolerance was the only strategy (Figure S5). Interestingly, however, the combined strategy generally resulted in parasite burdens similar to a pure resistance strategy (Figure S5), in spite of the majority of the immune response being allocated to tolerance (Figure 6). Precisely how this plays out in real hosts will depend on how effective their immune systems are; in our model, for simplicity, we have assumed that both resistance strategies and tolerance are equally efficacious, whereas in reality, it may be that an adult worm is much harder to clear than a larvae. However, this finding does suggest that measuring parasite burdens alone is insufficient to indicate the relative host investment in each strategy. Tolerance is often defined as the slope of condition against parasite burden (Read et al., 2008), but such a reaction norm could be skewed by hosts differentially investing in the two strategies. One approach that may be fruitful is gene‐knockout comparisons, such as in *D. melonogaster* (Gupta & Vale, 2017; Prakash et al., 2022); by removing specific mechanisms, one may be able to disentangle how each strategy is contributing to the host response to infection.

A combined strategy is clearly more than the sum of its parts. Our model predicted that hosts able to allocate their immune response between all three immune‐mediated strategies experienced substantially higher conditions (Figure 5) and reduced parasite burdens (Figure S5), and achieved this with a cheaper unit investment than tolerance alone (Figure S4). Interestingly, the major factor determining the success of a combined strategy was resource availability; there was little difference in attainable levels of condition across the different parasite trait scenarios explored. In all cases, however, alongside a strong tolerance response, hosts were predicted to also allocate resources to both resistance strategies (larval prevention and adult clearance) no matter which life stage was more virulent, albeit in differing amounts. This will also have population‐level benefits, reducing as it does the potential for onward transmission of the parasite. The importance of maintaining variation in host response can be seen in emerging evidence that parasite‐mediation strategies are parasite‐specific. For example, experiments in *D. melanogaster* have shown that mutations of a single gene yielded changes in both tolerance and resistance to bacteria; which of the two strategies changed, and in which direction compared to wild type, was dependent upon the specific microbial challenge (Ayres & Schneider, 2008). Consider also the differential responses of African buffalo to parasites of differing virulence, in which the less virulent *Cooperia* was tolerated but the more virulent *Haemonchus* resisted (Budischak et al., 2018).

The choice of parasite mitigation strategy will have profound consequences for a host, impacting their condition, survival and reproductive success. We have demonstrated that the efficacy of different strategies is highly dependent on timescale, parasite traits and resource availability. By combining different strategies, a host is able to exploit the benefits of each individual strategy, while minimising their downsides (e.g. immunopathology, or, to an extent, resource expenditure). This suggests that we will see all strategies being exploited, but that disentangling their contributions to host condition or parasite load may be difficult. However, model frameworks such as the one presented here that integrate environmental‐, host‐ and parasite‐related factors may help inform the collection and interpretation of empirical data, to understand how those drivers interact to shape host immune responses in natural systems.

## AUTHOR CONTRIBUTIONS

**Andrew D. Dean:** Conceptualization (equal); formal analysis (lead); investigation (lead); methodology (lead); project administration (equal); software (lead); validation (lead); visualization (lead); writing – original draft (lead); writing – review and editing (lead). **Dylan Z. Childs:** Funding acquisition (supporting); writing – review and editing (supporting). **Yolanda Corripio‐Miyar:** Writing – review and editing (supporting). **Mike Evans:** Writing – review and editing (supporting). **Adam Hayward:** Writing – review and editing (supporting). **Fiona Kenyon:** Funding acquisition (supporting); writing – review and editing (supporting). **Luke McNally:** Writing – review and editing (supporting). **Tom N. McNeilly:** Funding acquisition (supporting); writing – review and editing (supporting). **Robin J. Pakeman:** Funding acquisition (supporting); writing – review and editing (supporting). **Amy R. Sweeny:** Writing – review and editing (supporting). **Daniel H. Nussey:** Funding acquisition (lead); writing – review and editing (supporting). **Amy B. Pedersen:** Conceptualization (equal); formal analysis (supporting); funding acquisition (supporting); investigation (supporting); methodology (supporting); supervision (supporting); writing – original draft (lead); writing – review and editing (equal). **Andy Fenton:** Conceptualization (equal); formal analysis (supporting); funding acquisition (supporting); investigation (supporting); methodology (supporting); project administration (equal); software (supporting); supervision (lead); validation (supporting); visualization (supporting); writing – original draft (lead); writing – review and editing (lead).

## CONFLICT OF INTEREST STATEMENT

The authors declare no conflicts of interest.

## Supporting information


**Figure S1.** **–S10.**

## Data Availability

The R code used to run the simulations in this work is openly available on DRYAD at DOI:10.5061/dryad.59zw3r2gx.

## APPENDIX A Initial conditions

### A.1

We chose as initial conditions the parasite‐free state $\left(R,L,P,I,C\right)=\left(R_{0},0,0,I_{0},C_{0}\right)$, with

(A.1)
$$R_{0}=\frac{S_{R}}{r},\;I_{0}=\frac{{\mathrm{pS}}_{R}}{l}.$$

$R_{0}$ is simply the steady‐state solution to (2.1) with no parasites, and therefore no upregulation of I. $I_{0}$ represents a small fraction p of available resources allocated to the standing immunity, accounting for natural immune degradation l. Then, $C_{0}$ is given by the steady version of (2.5), namely

(A.2)
$$C_{0}=\frac{1}{b}\left(\frac{{\mathrm{aS}}_{R}}{w+h_{I}I_{0}}-1\right).$$

Note that the starting condition $C_{0}$ depends on resource availability $S_{R}$, and also on choice of strategy via the immunopathological harm $h_{I}$, which is only non‐zero for the prevention and clearance strategies.

## APPENDIX B Parameter selection

### B.1

The two parameters a and w encapsulate a range of physical and biological processes which combine to determine the condition of a host. For example, a host feeding on resources of poor quality or diverting resources to gestating or suckling offspring may be represented by reducing a, that is, the host has a reduced capacity to improve its condition. A host under significant energetic demands due to adverse weather conditions or the rigours of the rut may be represented by increasing w, that is, the host suffers from increased deterioration of condition. We were able to use (2.5) to check the parameter values used were sensible. Suppose that an initially well‐fed host, employing the anorexia strategy (i.e. I≡0), is starved and held in isolation from parasites. Then its resource level can be derived from (2.1) with $R\left(0\right)=\max\left(S_{R}\right)/r$ to give $R=\max\left(S_{R}\right)e^{-\mathrm{rt}}r$. Here $\max\left(S_{R}\right)$ is the maximum value of $S_{R}$ used in the current study. Then (2.5) is simply

(B.1)
$$\frac{dC}{dt}=\frac{a\max\left(S_{R}\right)e^{-\mathrm{rt}}}{1+\mathrm{bC}}-w.$$

Although this equation has no analytic solution, by checking at what time a host under such conditions dies, that is, when C reaches zero, we can confirm that the choice of a and w are sensible. Requiring that the host is initially alive, we impose $C_{0}>0$, and thus (7.2) gives

(B.2)
$$w<\left(a-\frac{h_{I}p}{l}\right)S_{R},$$

yielding an upper bound on baseline depletion rate of condition w. We chose our default values of a and w to yield a time to death of 10.1 days. We also chose parasite mortality to represent reasonable lifetimes for adults (≈100 days), and set larval mortality to be somewhat higher.
