# Supplementary material for: Host resources and parasite traits interact to determine the optimal combination of host parasite‐mitigation strategies
Source: Ecol Evol. 2024 Jun 19;14(6):e11310. doi: 10.1002/ece3.11310 (PMC11187858; doi:10.1002/ece3.11310)
Supplement: Supplementary file 1 — Figure S1. –S10. [file ECE3-14-e11310-s001.pdf]

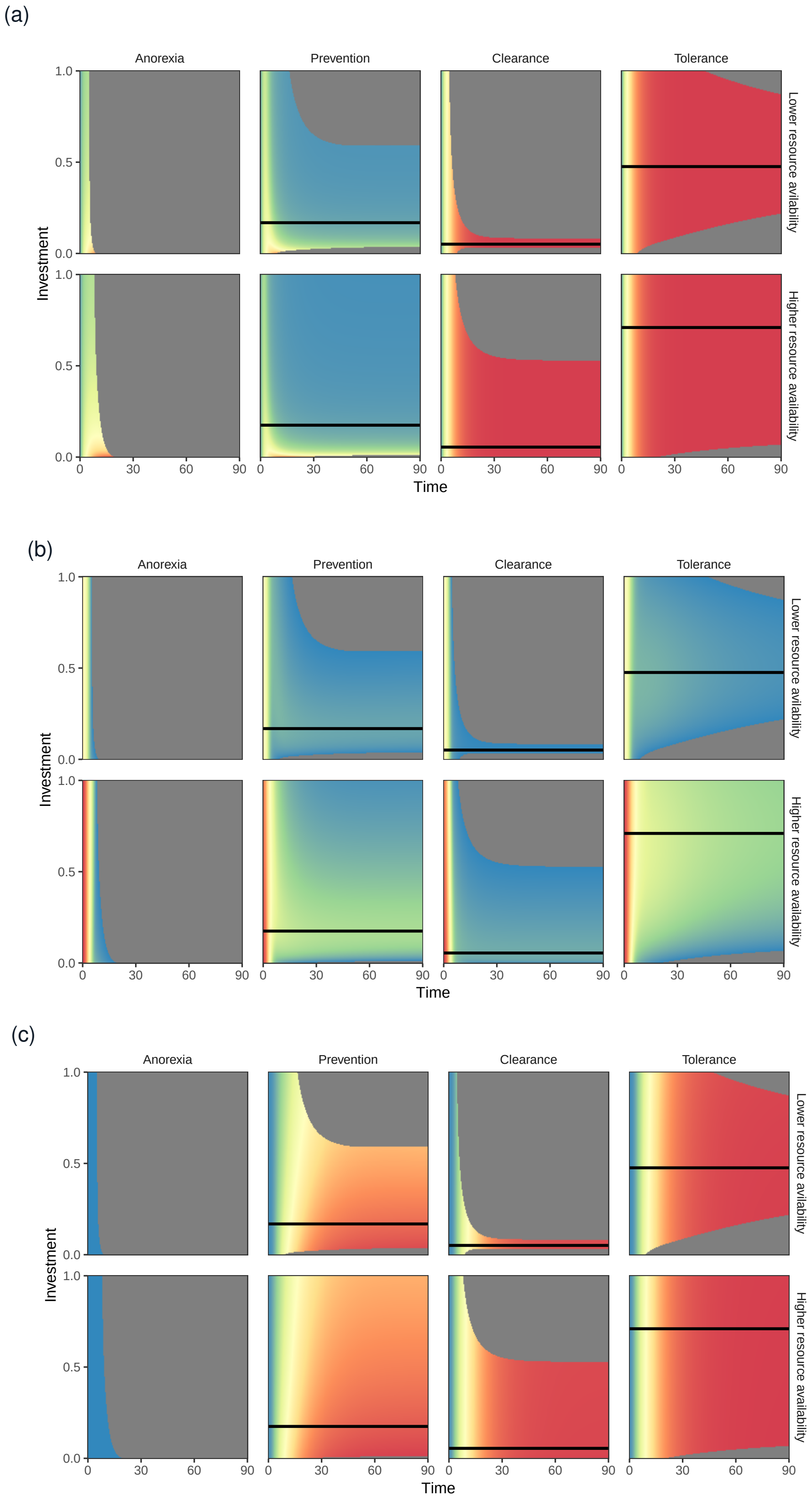

Figure S1: *Comparative dynamics (time on x-axis) of varying levels of investment (c, y-axis) in each of the four parasite-mitigation strategies (anorexia, larval parasite prevention, adult parasite clearance, tolerance) on A. larval parasite burden, B. host resource level and C. host immune response, under two different values of resource availability; low ($S_R = 3$; top rows) and high ($S_R = 5$; bottom rows). Parasite larvae and adults are assumed to be equally harmful ($h_L = h_P$ = 0.4); parasite maturation rate is set at $g = 0.1$; infection pressure is $S_L = 2$. The heat maps are scaled so values increase from blue to red; colours are normalised independently over each variable, so that the scale is different for host condition than for mature parasite load. Grey represents a dead host ($C(t) = 0$). There is no explicit immune response for anorexia, so the immune repsonse in this case is set to 0. Host condition and adult parasite burden are plotted in the main text, Figure 3.*

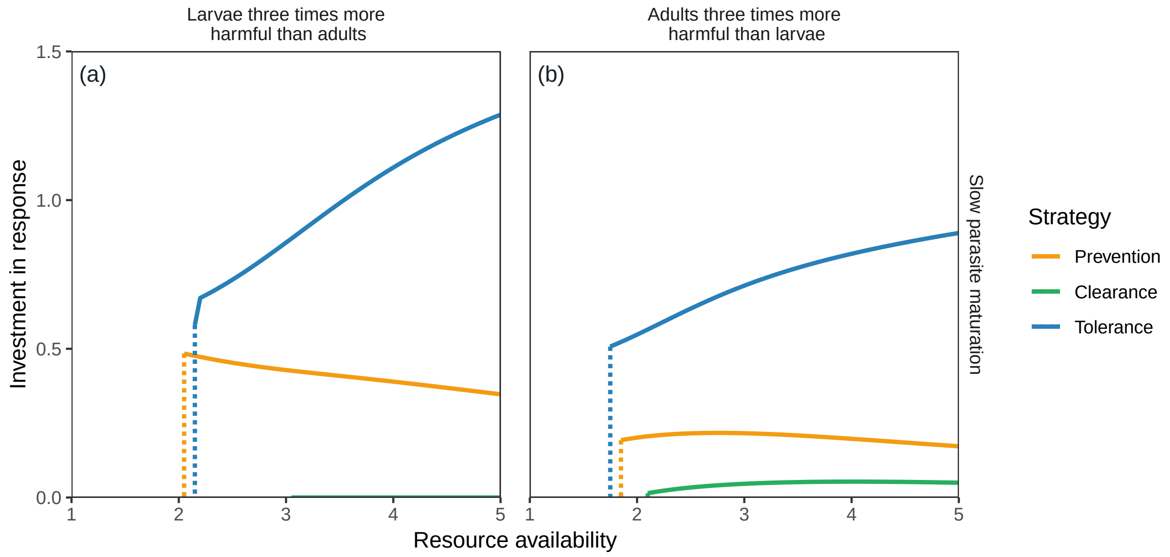

Figure S2: *Value of investment c that maximises mean condition achievable over one week ($t \in [0, 7]$), for each parasite-mitigation strategy, over a range of resource availability levels. (a) adults have higher virulence than larvae ($h_{\mathrm{L}} = 0.2, h_{\mathrm{P}} = 0.6$). (b) larvae have higher virulence than adults ($h_{\mathrm{L}} = 0.6, h_{\mathrm{P}} = 0.2$). Data are plotted only for those parameter values for which the hosts survives; dashed vertical lines indicate the minimum value of S_{R} at which the host survives. Anorexia is omitted, as in this context the optimum strategy is starvation, i.e $k_{\mathrm{A}} = \infty$. In (b), any investment in the clearance strategy decreases host condition, hence $c = 0$ for this strategy.*

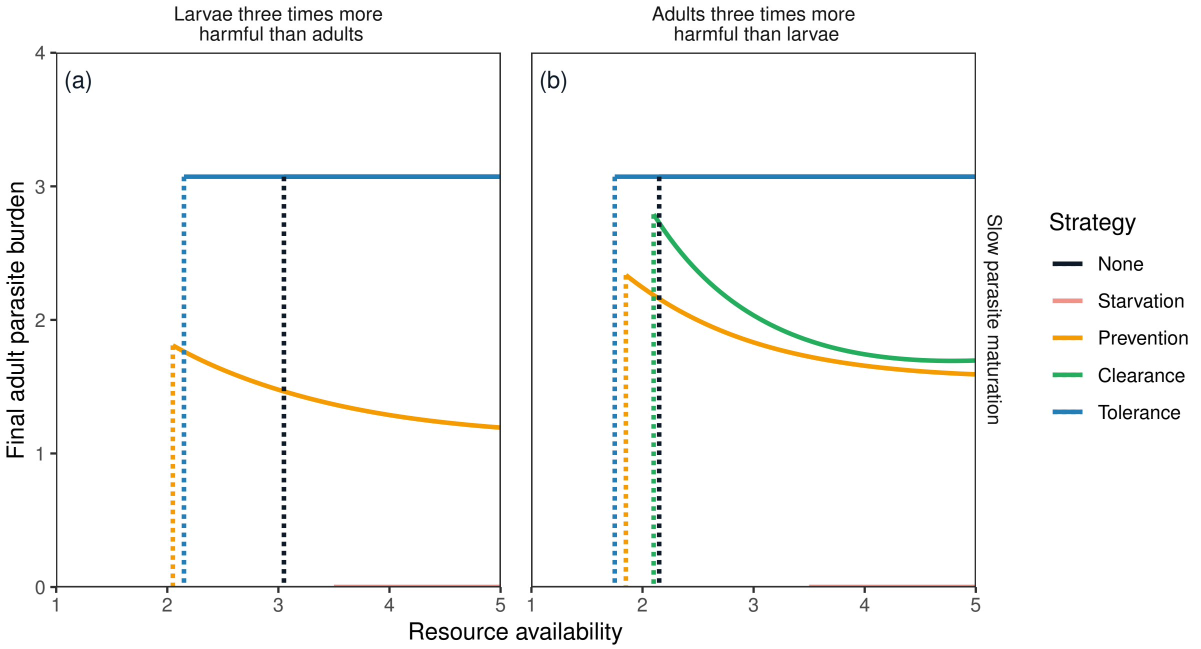

Figure S3: *Final adult parasite burden* P *corresponding to the investment that maximises mean condition over one week* ($t \in [0, 7]$)*, for each parasite-mitigation strategy, over a range of resource availability levels. (a) adults have higher virulence than larvae* ($h_{\mathrm{L}} = 0.2, h_{\mathrm{P}} = 0.6$)*. (b) larvae have higher virulence than adults* ($h_{\mathrm{L}} = 0.6, h_{\mathrm{P}} = 0.2$)*. Data are plotted only for those parameter values for which the hosts survives; dashed vertical lines indicate the minimum value of* S_{R} *at which the host survives. In (b), any investment in the clearance strategy decreases host condition, i.e. is equivalent to no strategy.*

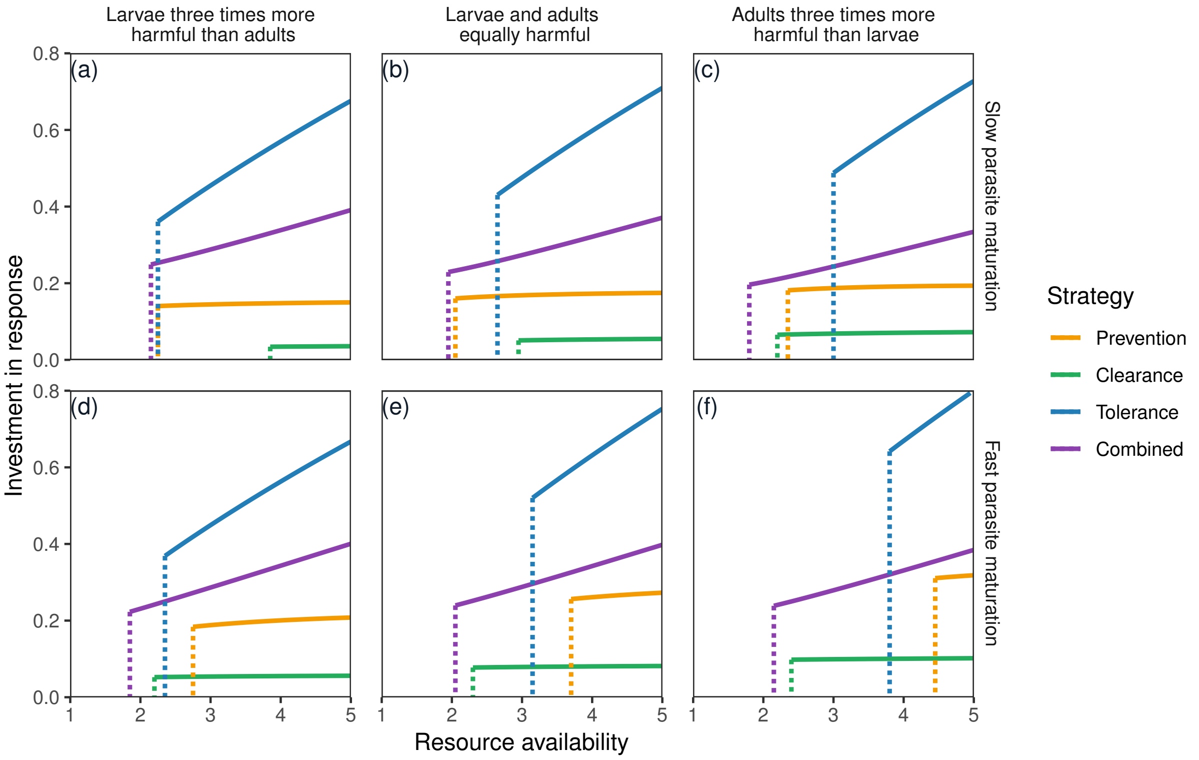

Figure S4: *Value of investment c that maximises mean condition achievable over one season ($t \in [0, 90]$), for each parasite-mitigation strategy, alone and combined, for a range of resource availability levels. Left column: adults have higher virulence than larvae ($h_\mathrm{L} = 0.2, h_\mathrm{P} = 0.6$). Centre column: adults and larvae have equal virulence ($h_\mathrm{L} = 0.2 = h_\mathrm{P} = 0.4$). Right column: adults have higher virulence than larvae ($h_\mathrm{L} = 0.6, h_\mathrm{P} = 0.2$). Top row: parasites mature relatively slowly ($g = 0.1$). Bottom row: parasites mature relatively quickly ($g = 0.5$). Data are plotted only for those parameter values for which the host survives; dashed vertical lines indicate the minimum value of S_R at which the host survives. The anorexia strategy or no strategy do not appear in any panel, as both choices lead to host death over this time period.*

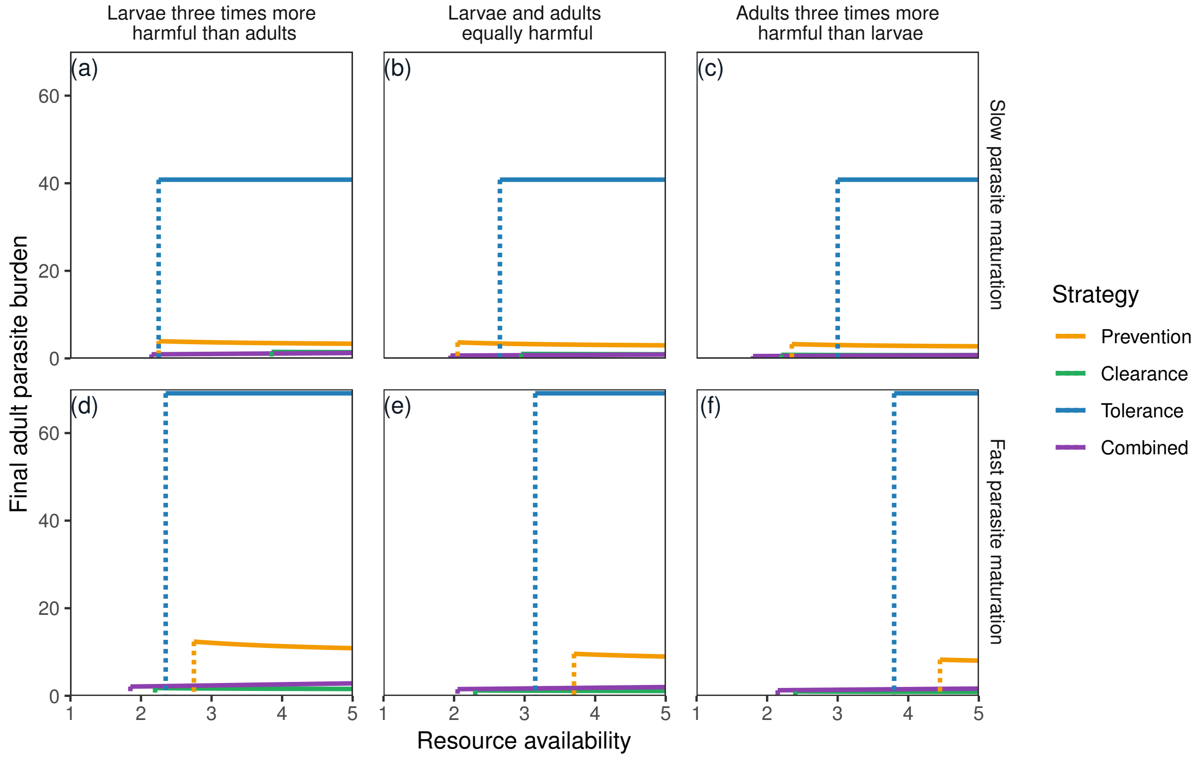

Figure S5: *Final adult parasite burden P corresponding to the investment that maximises mean condition over one season ($t \in [0, 90]$), for each parasite-mitigation strategy, alone and combined, for a range of resource availability levels. Left column: adults have higher virulence than larvae ($h_{\mathrm{L}} = 0.2, h_{\mathrm{P}} = 0.6$). Centre column: adults and larvae have equal virulence ($h_{\mathrm{L}} = 0.2 = h_{\mathrm{P}} = 0.4$). Right column: adults have higher virulence than larvae ($h_{\mathrm{L}} = 0.6, h_{\mathrm{P}} = 0.2$). Top row: parasites mature relatively slowly ($g = 0.1$). Bottom row: parasites mature relatively quickly ($g = 0.5$). Data are plotted only for those parameter values for which the host survives; dashed vertical lines indicate the minimum value of S_{R} at which the host survives. The anorexia strategy or no strategy do not appear in any panel, as both choices always leads to host death over this time period.*

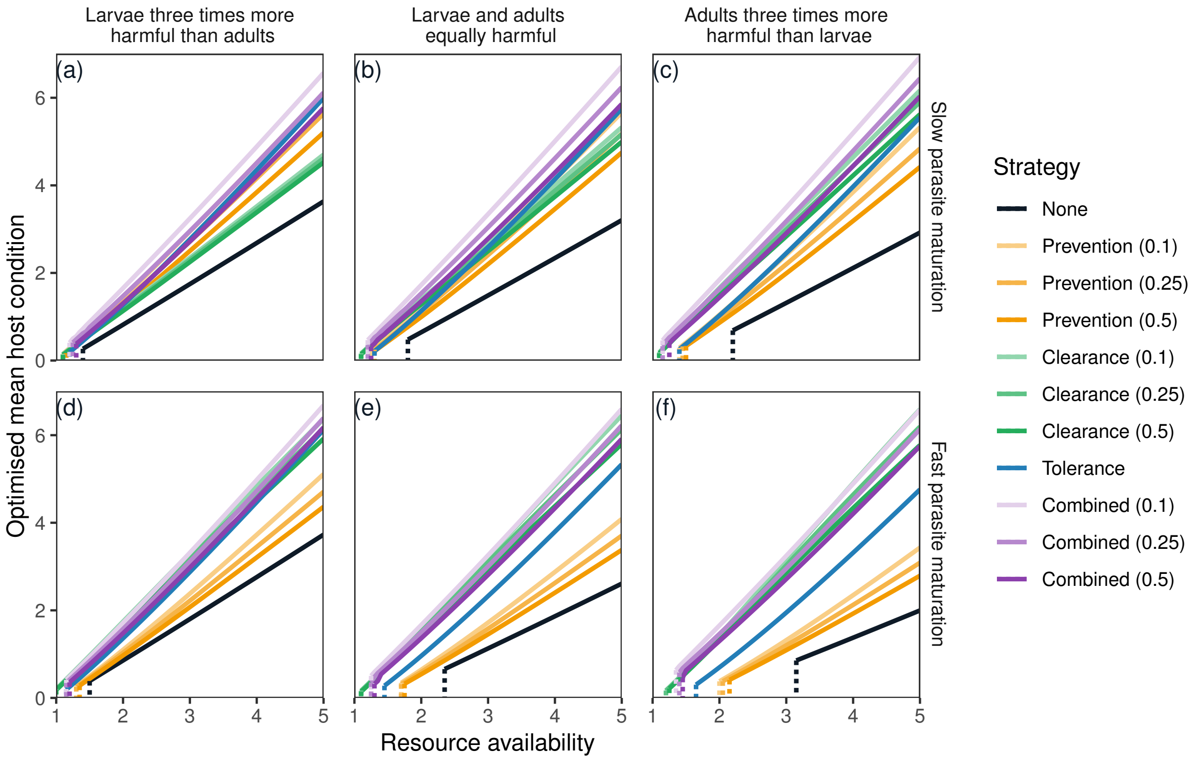

Figure S6: *Long-term maximum mean condition over one season ($t \in [0, 90]$), for each parasite-mitigation strategy, alone and combined, for a range of resource availability levels and infection pressure $S_L = 0.5$ ($S_L = 2$ in main text Figure 5). Three different values of the immunopathology parameters $h_{I,0}$ and $h_{I,1}$ are shown, indicated by the numbers in brackets in the figure legend. Left column: adults have higher virulence than larvae ($h_L = 0.2, h_P = 0.6$). Centre column: adults and larvae have equal virulence ($h_L = 0.2 = h_P = 0.4$). Right column: adults have higher virulence than larvae ($h_L = 0.6, h_P = 0.2$). Top row: parasites mature relatively slowly ($g = 0.1$). Bottom row: parasites mature relatively quickly ($g = 0.5$). Data are plotted only for those parameter values for which the host survives; dashed vertical lines indicate the minimum value of S_R at which the host survives. The anorexia strategy or no strategy do not appear in any panel, as both choices always leads to host death for these parameters over this time period.*

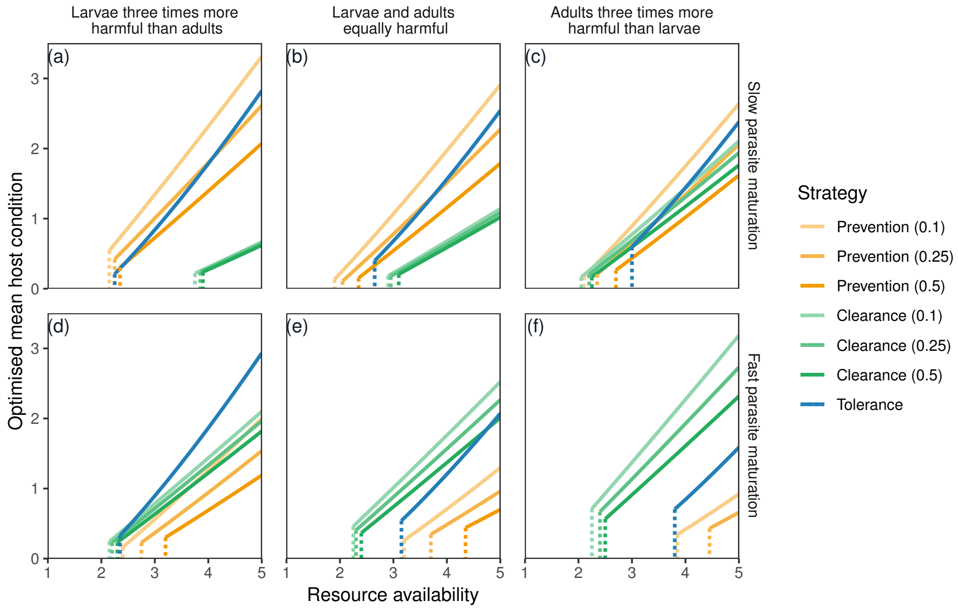

Figure S7: *Long-term maximum mean condition over one season ($t \in [0, 90]$), for each parasite-mitigation strategy, alone and combined, for a range of resource availability levels and infection pressure $S_\mathrm{L} = 2$. Three different values of the immunopathology parameters $h_{\mathrm{I},0}$ and $h_{\mathrm{I},1}$ are shown, indicated by the numbers in brackets in the figure legend. Left column: adults have higher virulence than larvae ($h_\mathrm{L} = 0.2, h_\mathrm{P} = 0.6$). Centre column: adults and larvae have equal virulence ($h_\mathrm{L} = 0.2 = h_\mathrm{P} = 0.4$). Right column: adults have higher virulence than larvae ($h_\mathrm{L} = 0.6, h_\mathrm{P} = 0.2$). Top row: parasites mature relatively slowly ($g = 0.1$). Bottom row: parasites mature relatively quickly ($g = 0.5$). Data are plotted only for those parameter values for which the host survives; dashed vertical lines indicate the minimum value of S_R at which the host survives. The anorexia strategy or no strategy do not appear in any panel, as both choices always leads to host death for these parameters over this time period.*

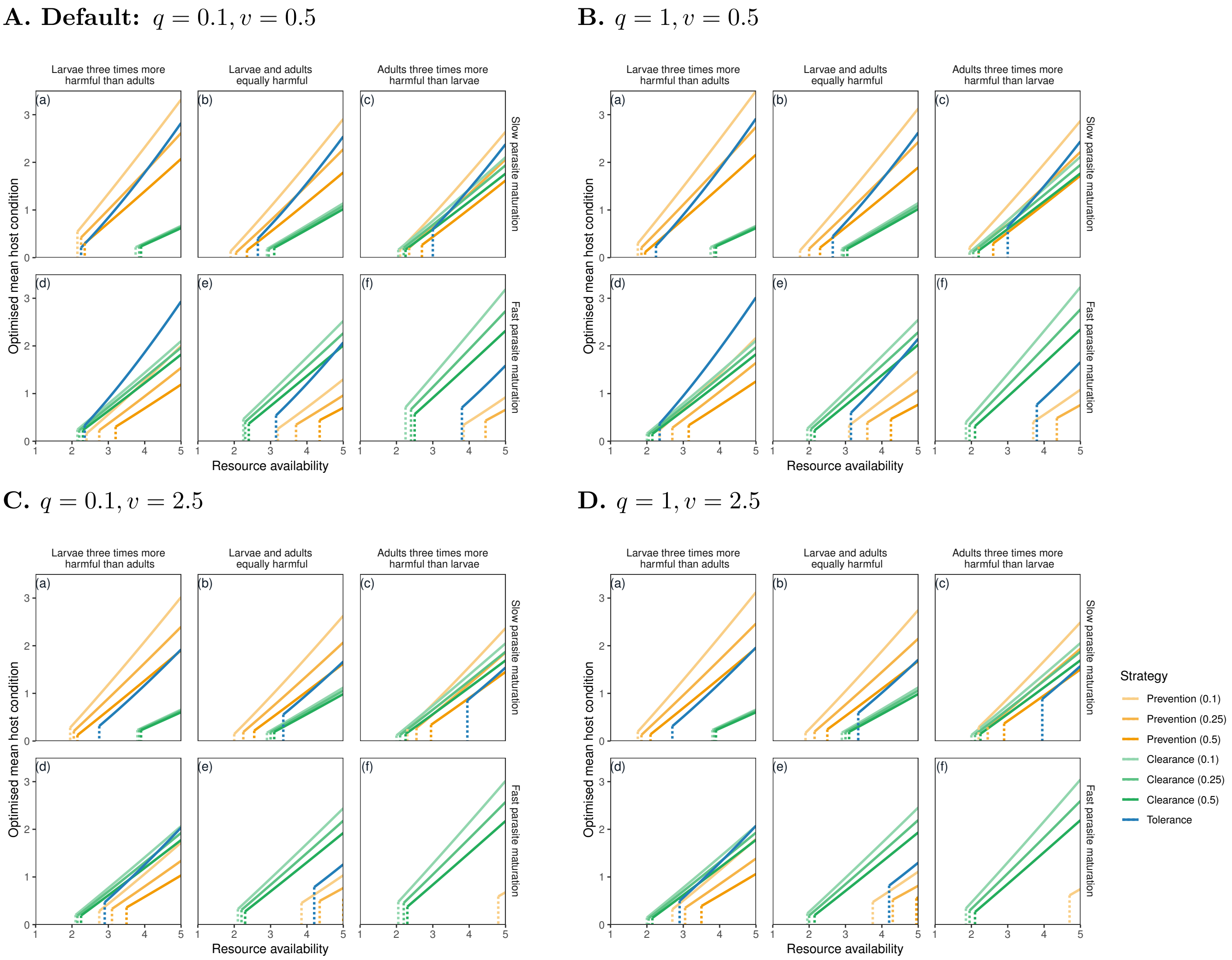

Figure S8: *Comparing the effects of the immune response production parameters. q represents how fast the immune response is produced, v how rapidly it saturates.*

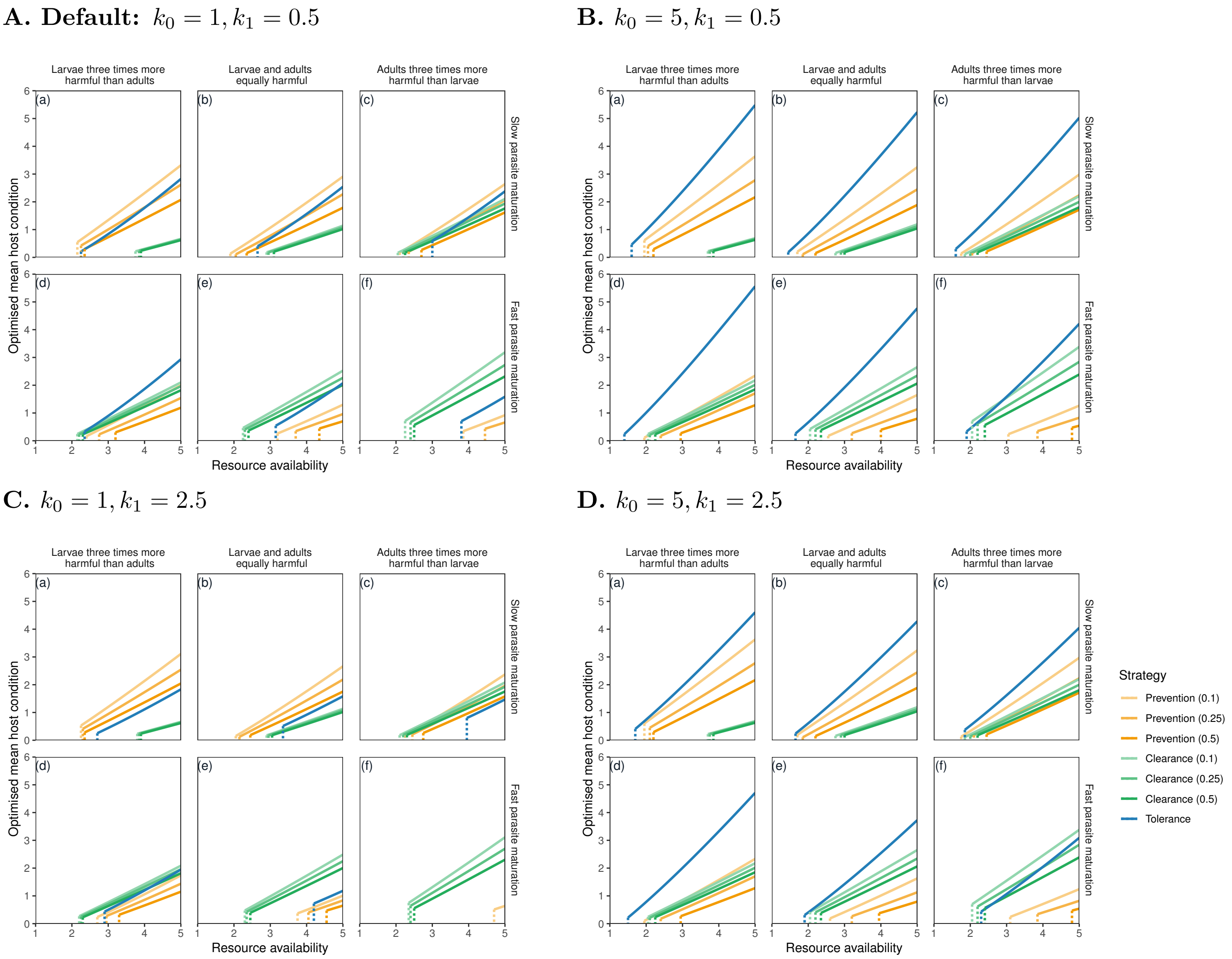

Figure S9: *Comparing the effects of the relationship between unit investment c and immune strength k.* k_0 *represents the maximum strength,* k_1 *how rapidly the relationship saturates.*

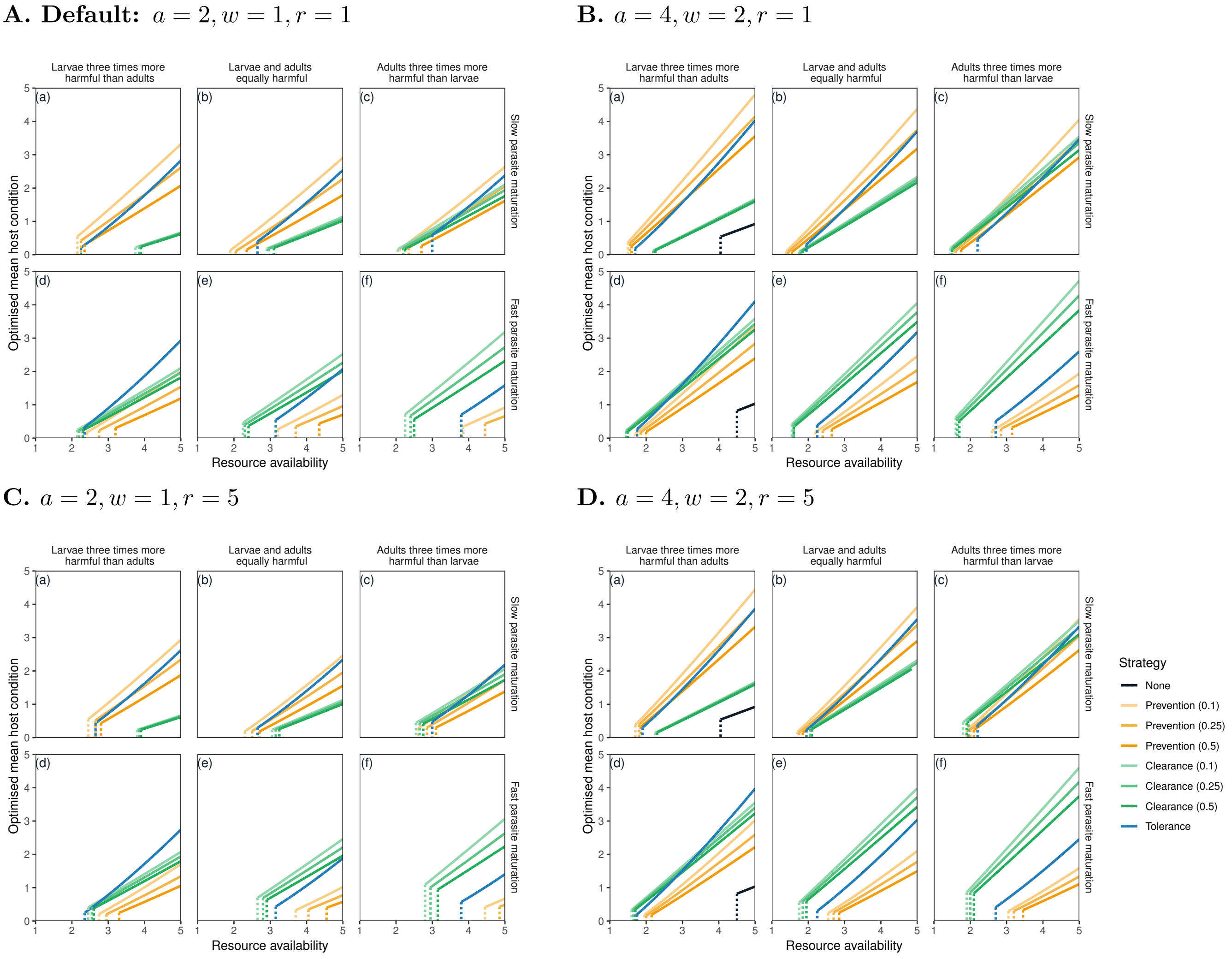

Figure S10: *Comparing the effects of resource processing and condition.* a *represents the rate of condition increase,* w *the condition loss,* r *the rate of resource processing. With default values of* a *and* w*, an initially well-resourced (*$S_R = 5$*; cf. Appendix B) survives for 10.1 days when starved and parasite-free; when* $a = 4, w = 2$*, a host in the same situation survives for 5.7 days.*
